# The Effect of Environmental Stressors on Tinnitus: A Prospective Longitudinal Study on the Impact of the COVID-19 Pandemic

**DOI:** 10.3390/jcm9092756

**Published:** 2020-08-26

**Authors:** Winfried Schlee, Sondre Hølleland, Jan Bulla, Jorge Simoes, Patrick Neff, Stefan Schoisswohl, Stella Woelflick, Martin Schecklmann, Axel Schiller, Susanne Staudinger, Thomas Probst, Berthold Langguth

**Affiliations:** 1 Department of Psychiatry and Psychotherapy, Bezirksklinikum, University of Regensburg, 93053 Regensburg, Germany; jan.bulla@uib.no (J.B.); jp.simoes@live.com (J.S.); patrick.neff@ukr.de (P.N.); stefan.schoisswohl@ukr.de (S.S.); stella-ainikki.woelflick@stud.uni-regensburg.de (S.W.); martin.schecklmann@medbo.de (M.S.); axel.schiller@medbo.de (A.S.); susanne.staudinger@medbo.de (S.S.); berthold.langguth@medbo.de (B.L.); 2 Department of Mathematics, University of Bergen, 5020 Bergen, Norway; sondre.hoelleland@hi.no; 3 Institute of Marine Research, 5817 Bergen, Norway; 4 URPP “Dynamics of Healthy Ageing”, University of Zürich, 8006 Zürich, Switzerland; 5 Department for Psychotherapy and Biopsychosocial Health, Danube University Krems, 3500 Krems, Austria; thomas.probst@donau-uni.ac.at

**Keywords:** tinnitus distress, personality traits, big five, stress, coronavirus, SARS-CoV-2, quarantine, pandemic, COVID-19, social isolation

## Abstract

Tinnitus, the perception of sound in the absence of a corresponding sound, and the distress caused by it, is rarely a static phenomenon. It rather fluctuates over time depending on endogenous and exogenous factors. The COVID-19 pandemic is a potential environmental stressor that might influence the individually perceived tinnitus distress. Since not all people are affected by the pandemic in the same way, the situation allows one to identify environmental factors and personality traits that impact tinnitus distress differently. In our study, 122 tinnitus patients were included at two time points: in the year 2018 and during the German lockdown in April 2020. We assessed tinnitus-related distress, depressive symptoms, personality characteristics and the individual perception of the pandemic situation. On average, there was only a small increase of tinnitus distress with heterogeneous changes during the lockdown. People perceiving the situation as generally stressful with increased grief, frustration, stress and nervousness reported the worsening of tinnitus distress. People with high values in neuroticism also reported the worsening of tinnitus distress, while the personality traits extraversion, conscientiousness and openness seemed to be a protection factor. The study identifies factors that influence tinnitus distress change during a pandemic and spots those patients that need specific help in the pandemic situation.

## 1. Introduction

Tinnitus, the perception of sound in the absence of a corresponding stimulus, is highly prevalent, and frequently associated with emotional distress, depressive symptoms, anxiety and insomnia [1,2,3]. The most relevant risk factor for the development of tinnitus is hearing loss, and it is assumed that the emergence of tinnitus is a reflection of brain mechanisms that aim to compensate for the lack of auditory input [4,5]. However, not everybody with hearing loss develops tinnitus. Further factors such as altered muscle tension in the neck or temporomandibular joint disorders can contribute to the development of tinnitus [6,7], illustrating the etiological heterogeneity of tinnitus.

In addition, there is a strong and complex bidirectional interaction between tinnitus and stress. Many tinnitus patients perceive their tinnitus as stressful, intrusive and annoying, and a considerable subgroup develop insomnia, attentional and psychological problems, like depression or anxiety, as a consequence of the ongoing tinnitus perception [8,9,10]. A bidirectional interaction is suggested, since stress can also cause or aggravate tinnitus perception. Many patients report that the onset of their tinnitus was preceded by stressful events [11,12,13], and prospective studies have revealed that patients with depression have an increased risk to develop tinnitus [14]. Moreover, the stress level and the affective state mediate the relationship between the loudness of the tinnitus and the individually perceived distress of tinnitus [15]. In addition, dynamics of emotions are associated with the course of tinnitus over time [16], which adds to the complex interaction of tinnitus distress, tinnitus loudness, stress and emotional perception.

Among the main coping strategies in tinnitus to regulate stress and difficult emotions are social and physical activation, as well as strategies distracting from the annoying sound [17]. We therefore hypothesize that any environmental, social, societal and occupational factors that prevent tinnitus patients to apply these coping strategies will lead to an increase of tinnitus distress. The environmental and societal factors during the pandemic of the novel coronavirus disease 2019 (COVID-19) with the governmental lockdown are measures that can potentially limit the coping strategies of tinnitus patients and lead to an increase of tinnitus distress.

Furthermore, it has been demonstrated that personality characteristics are also associated with the perception of tinnitus distress. Specifically, higher degrees of neuroticism have been repeatedly shown to be associated with higher tinnitus severity [18,19,20]. However, the details of the interaction between personality characteristics and tinnitus are not understood yet. Based on these earlier studies, we hypothesized that lower degrees of neuroticism might be a protection factor for tinnitus patients during the COVID-19 pandemic.

In March 2020, the Federal Government of Germany announced, similar to most other countries, an almost complete shutdown of public life, with the closure of schools, universities, restaurants, shops etc., with the goal to slow down the spread of the severe acute respiratory syndrome coronavirus 2 (SARS-CoV-2). Such a complete shut-down of public life, together with imposed isolation, is expected to have a substantial impact on many aspects of people’s lives, causing considerable psychological strain, and triggering a variety of psychological problems [21]. A recent review of the literature on the psychological effects of quarantine during past epidemics and pandemics highlighted that quarantined people are more likely to show psychological distress [22] and have an increased prevalence of psychological symptomatology, including post-traumatic and depressive symptoms, stress, and anxiety [23,24]. This notion is confirmed by studies that investigated the impact of the COVID-19 emergency, and which suggest that about 30–50% of the investigated people experience psychological distress [25,26]. Interestingly, the unique situation of the COVID-19 pandemic, with an almost complete public shutdown, did not cause distress in all people. Regular surveys investigating the psychological state of the German population during the coronavirus crisis revealed that the percentage of people who consider themselves as happy increased during the corona pandemic [27]. Taken together, it was expected that the individual reports about distress were to be heterogeneous among the study population. Such a variance in the individual responses would even be beneficial for the analysis of meaningful associations.

With this study, we wanted to investigate the impact of the coronavirus pandemic on subjectively perceived stress and individual tinnitus-related distress. More specifically, we wanted to know whether tinnitus complaints increase during such a collective and potentially stressful situation, and whether this effect depends on personality factors, specific quarantine related burdens or individual reactions to these burdens.

To address these questions, we conducted a survey in April 2020, during the corona-related lockdown in Germany. This survey consisted of questions regarding tinnitus complaints, personality traits, quarantine-related burdens and depressive symptoms. Thereby, we could refer to a sample of patients who visited our tinnitus clinic (Regensburg, Germany) between 2012 and 2017, and who participated in a follow-up investigation in 2018 [20]. The availability of this data from 2018 enabled us to compare the results during the coronavirus pandemic with the situation before the outbreak of the disease.

## 2. Methods

### 2.1. Study Design, Sample and Measures

All patients who attended the multidisciplinary tinnitus clinic of the University of Regensburg (Regensburg, Germany) between 2012 and 2017 were invited in 2018 to participate in a survey about their tinnitus [20]. Overall, 388 of 1213 contacted patients completed several questionnaires, including the German versions of the Tinnitus Handicap Inventory (THI) [28], the Tinnitus Questionnaire (TQ) [29], the Major Depression Inventory (MDI) [30] and the Big Five Index 2 (BFI2) [31]. Patients were also asked whether they were willing to participate in additional online-based surveys, to which *n* = 244 agreed. These patients were contacted by email on 14th April 2020, and asked to complete an online survey, which included the THI, mini-TQ, MDI, BFI-2, and a newly developed questionnaire for assessment of the burden of quarantine, named the Social Isolation Electronic Survey (SOISES). The 37 items of the SOISES had to be answered on a 5-point Likert scale with the endpoints “entirely true” and “entirely wrong”. The items were based on a recent meta-analysis of the psychological strains of quarantine [22], and are given in the supplementary material Tables S1 and S2. Notably, the survey in 2018, which served as the baseline for this study, was completed in a paper and pencil version, whereas the survey during the pandemic of 2020 was completed as a completely electronic survey. This online survey was conducted utilizing the Tinnitus Database (www.tinnitus-database.de), hosted by the University Regensburg (ethical approval number 18-1041-101). During the pandemic, the participants received a weblink via email, leading them to the tinnitus database to fill in the questionnaires electronically within a time window of 7 days. The main regulations during the lockdown period in this region were restrictions of personal contact outside the household, short-time work in many work sectors, travel restrictions, the closing of shops that are not vital, and restrictions to leave the house or apartment, with the exception of grocery shopping, going for a walk, and low-risk individual sport activities.

### 2.2. Statistical Analysis

Statistical analyses were conducted in R [32], using R-version 3.6.3 (29 February 2020). Parametric (*t*-test) and non-parametric tests (Wilcoxon-test) were calculated to analyze the difference between the two time points. Cohen’s D and Wedge’s G effect size were calculated using the effsize package [33], which implements the approach suggested by Gibbons et al. [34] The routine also uses a correction that takes the correlation of the two samples into consideration [35]. The method of Benjamini and Yekutieli [36] served for adjusting *p*-values for multiple comparisons. Correlation analysis was done using Spearman correlations, and a bootstrap method for calculating confidence intervals. The timepoint of the baseline measure in 2018 was used for the correlation analysis between personality characteristics and change of tinnitus distress.

## 3. Results

### 3.1. Participants

In total, 122 chronic tinnitus patients (42 female, 80 male) with an average age of 54.0 (SD 10.9) years at baseline participated in the study. At baseline, the average duration of tinnitus was 160.9 (SD 99.6) months. More details about the study group at baseline are given in Table 1.

The baseline assessments were done between 28 March 2018 and 20 August 2018. The assessments during the COVID-19 pandemic were done between 14 April 2020 and 29 April 2020. On average, there were 685.05 (±29.8 SD) days between the two assessments.

### 3.2. Average Influence of the Pandemic Situation

**Figure 1:**
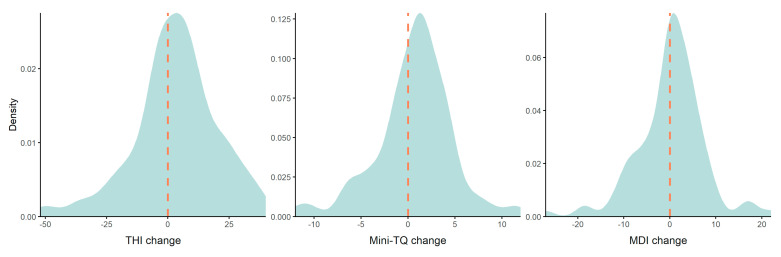
Influence of pandemic situation on tinnitus-related distress and depressive symptoms. The difference (change) was calculated by subtracting time point 2 (pandemic situation) from time point 1 (baseline). Higher values indicate more severe symptoms during the pandemic.

The direct comparison of the tinnitus questionnaires before and during the pandemic revealed an increase of the THI and Mini-TQ scores during the pandemic (see Table 2 and Figure 1). There was no significant change in the MDI score. Among the personality traits, there was a significant reduction of the score of “neuroticism”. The other personality domains remained unchanged.

### 3.3. Influence of Quarantine-Related Burdens on Tinnitus

**Figure 2:**
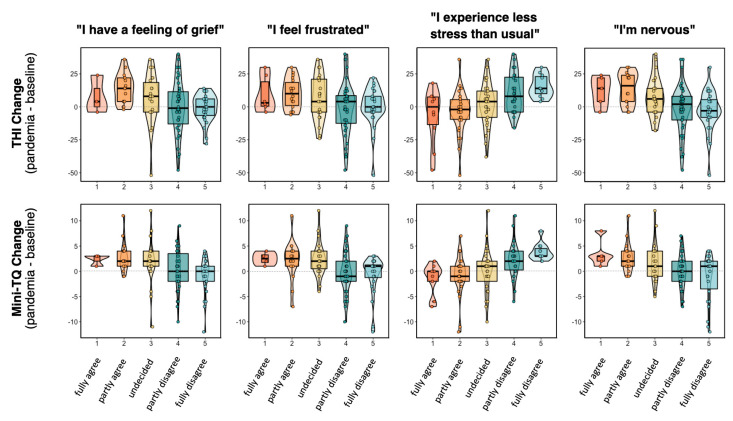
Relationship between pandemic-related burden and change of tinnitus distress. Change of tinnitus distress was calculated by subtracting time point 2 (pandemic situation) from time point 1 (baseline).

Items of the SOISES questionnaire that correlate significantly with the change in THI score or with the change in Mini-TQ score were related to feelings of grief, frustration, stress and nervousness, and are shown in Table 3 and in Figure 2. The complete table with all correlation analyses is given in the supplemental material Tables S1 and S2.

### 3.4. Influence of Personality Factors on Tinnitus Change

Correlation analyses between the BFI2 domains during the pandemic situation and changes in the tinnitus scores (THI and Mini-TQ) revealed that the domain “neuroticism” of the BFI2 correlated significantly with both the THI and the Mini-TQ change score. The higher the score for “neuroticism”, the more pronounced was the worsening of the tinnitus. In contrary, the personality factors “extraversion”, “conscientiousness”, and “openness” correlated negatively with the change in THI or Mini-TQ. Higher values in these personality factors were related to an improvement of tinnitus-related distress (see Table 4 and the scatterplots in Supplemental Material Figures S1 and S2).

## 4. Discussion

In this study, we investigated the effects of the COVID-19 pandemic situation on tinnitus patients. For this purpose, we contacted patients who had previously attended the Tinnitus Clinic of the University of Regensburg, and who had participated in a survey regarding their tinnitus in 2018. Thus, the tinnitus diagnosis had been confirmed by a clinical investigation in all patients. Moreover, the availability of the assessments in 2018 enabled a prospective longitudinal study design with a direct within-subject comparison of the situation, before and during the pandemic. In addition to tinnitus severity and handicap, we assessed depressive symptoms, personality traits, and potential quarantine-related complaints. The study revealed new insights on environmental influences on tinnitus distress.

### 4.1. Change of Tinnitus Distress

We observed a small increase of tinnitus distress in both questionnaires, the THI and the Mini-TQ, which was in line with the main hypothesis of this investigation. On average, the THI score increased by 4.37 points. The average increase of the Mini-TQ is 0.97 points. This increase appeared to be statistically significant, however, the effect sizes were small (Cohen’s D = 0.13, Hedge’s G = 0.129), and the effects were not statistically significant after p-value adjustment for multiple comparisons. This increase of tinnitus distress is also in line with a recent report on 16 chronic tinnitus patients during the Italian lockdown. In this sample, 75% (12 out of 16) of the patients reported an increase of tinnitus distress by one grade in the THI (mild to moderate in 9 cases, moderate to severe in 3 cases) [37]. The authors hypothesize that the absence of environmental masking sounds may enhance the conscious perception of the tinnitus sound.

As shown in Figure 1, the change of tinnitus distress in the pandemic situation was heterogeneous; a considerable number of patients reported an increase of tinnitus distress, while others reported a decrease of tinnitus distress. This heterogeneous response was not surprising, and allowed more specific analysis on the perception of the subjective perception of the pandemic situation, with the focus to identify which aspects of the situation relate to the change of tinnitus distress. Moreover, we examined whether neuroticism, as well as other personality traits, influence the change of tinnitus distress. Results of these more specific analyses are discussed below.

Given the interval of almost two years between the two time-points of tinnitus assessment, we cannot entirely disentangle to which extent the observed changes are related to the current pandemic, and to which extent they merely reflect unspecific changes over time. Moreover, the questionnaires were completed in a paper and pencil version in 2018, and in an electronic form in 2020. Repeated assessments of the same sample over time might help to approach this question in future studies. Such an approach could also capture varying individual emotional reactions during the course of the pandemic. Emotional variations are expected, as the pandemic situation and its impact on society are highly dynamic. Our survey was performed relatively soon after the beginning of the lockdown, and the answers might be different after a longer duration of the lockdown, or during the way back towards normality. However, it is highly unlikely that the worsening of tinnitus scores during the pandemic is merely an unspecific effect of time, as the previous investigation of the same sample revealed an improvement of tinnitus scores over time [20].

### 4.2. Relationship of Tinnitus Change to the Subjective Perception of the Pandemic Situation

The change of the tinnitus distress scores was significantly correlated to four items in the SOISES questionnaire, which was designed to assess the subjective perception of the pandemic situation. The four items were related to grief (“I have a feeling of grief”), frustration (“I feel frustrated”), stress (“I experience less stress than usual”, inverse relationship) and nervousness (“I’m nervous”). Only items that survived adjustments for multiple comparisons with at least one of the two tinnitus questionnaires are reported in Table 3 and Figure 2. Correlations with the remaining items are reported in the Supplemental Material Table S1. Tinnitus patients who perceived grief, frustration, stress and nervousness during the pandemic situation reported an increase of tinnitus-related distress. Interestingly, these items asked for rather generic aspects of the pandemic situation. Items that focused on more specific pandemic-related worries (e.g., health-related, financial), or on the objective situation (e.g., social isolation, use of social media, impact of the pandemic on professional activities), were not significantly correlated to tinnitus changes. It is well known from clinical experience that stressful life events can worsen tinnitus distress. These findings of this study suggest that the worsening of tinnitus-related distress is mainly related to a general emotional reaction during the pandemic. It remains to be shown if this finding can be generalized to other threatening situations.

From a clinical perspective, this result might hint at specific psychological interventions for severe cases of tinnitus increase during the pandemic. As personal contacts are highly restricted during a pandemic, online based interventions should be mainly considered. Internet-based cognitive-behavioral therapy is well established in the management of tinnitus [38,39]. One could also speculate that mindfulness-based stress reduction (MBSR) interventions might represent a promising approach to better cope with negative emotional reactions during a pandemic situation [12,40]. Online-based MBSR is available, but has not yet been systematically investigated in tinnitus patients.

### 4.3. No Statistically Significant Change of Depressive Symptoms

Interestingly, there was no significant change in depressive symptoms as measured by the MDI (*p* > 0.5). The Cohen’s D and Hedge’s G effect size was below 0.1, indicating no change of depressive symptoms. This is in contrast to other studies comparing depression, before vs. in COVID-19 (e.g., [41]).

### 4.4. Change of Personality Characteristics

The BFI2 questionnaire revealed a reduction of the average neuroticism during the pandemic situation. The reduction of neuroticism was an unexpected finding, as our study did not focus primarily on personality changes during the pandemic. Furthermore, changes in the mean scores were considerably low, which only allows for cautious interpretations. One could speculate whether the situation of a global thread, requiring a common effort and thus also creating a sense of community and a common goal, as well as an openness to rethink established procedures in society, might reduce neuroticism. This fits with results from some surveys that indicated a mean increase in happiness and satisfaction during the lockdown situation in Germany in Spring 2020 [27].

### 4.5. Relationship of Tinnitus Change to Personality Traits

The correlation analyses between changes in tinnitus scores and personality traits are linked to a well-known association between neuroticism and tinnitus severity. Increased neuroticism has been found to be associated with higher tinnitus severity [19,42], and also predicts the worsening of tinnitus over time [20]. Our analysis now revealed that this relationship also holds true during the pandemic. Furthermore, the data suggest that the personality factors “extraversion”, “conscientiousness”, and “openness” might be personality traits that protect the individual against the worsening of the tinnitus-related distress in exceptional and unexpected situations like the COVID-19 pandemic. Interestingly, the personality factors extraversion and conscientiousness have also been found to correlate with the engagement of containment measures during the pandemic in a Brazilian population. People with high scores in extraversion are associated with lower compliance of social distancing rules, while people with high scores in conscientiousness are associated with higher compliance of social distancing and handwashing rules [43]. So far, it is not clear if this result can be generalized to other countries as well, since the pandemic situation, infection rates and governmental measures, vary largely between different countries. Nevertheless, this result gives a hint on the complex interaction between tinnitus, environmental stressors and personality factors. Personality factors might influence the behavioral reaction to the environmental stressor, which also interacts with the perception of the tinnitus sound and tinnitus-related distress. This further adds to an explanation of the heterogeneous responses that we observed in the study, and encourages further studies on the interaction of stressors, personality factors and tinnitus distress.

### 4.6. Heterogeneous Responses

The results of this study demonstrate that the individual reactions to the pandemic varied largely among the investigated sample, and even if there was a mean increase of the THI and the Mini-TQ, there were many patients with clearly lower tinnitus scores during the pandemic. This observed heterogeneity of the individual course of the tinnitus during the pandemic further supports that it is of utmost importance to better identify and understand the factors that contribute to the worsening or improvement of subjective tinnitus severity over time [15,16,20,44].

In this context, further studies will also need to include analyses on the medication taken to counteract the COVID-19 symptoms. For instance, a recent mini-review highlighted the potential ototoxic side effect of hydroxychloroquine, a medication that is currently discussed as a treatment for COVID-19 [45]. The side effects might increase with higher dosage. Moreover, there are more studies need to investigate the long-term effects of a coronavirus infection on the hearing system and tinnitus. Currently, there is only a limited number of research papers with a small sample size available [46].

## Figures and Tables

**Table 1:** Description of the study group. Ratio or mean values are given ± standard deviation.

	Ratio/Mean [SD]
Gender ^1^	42/80
age	54.0 [10.9]
tinnitus duration [months]	160.9 [99.6]
laterality of tinnitus ^2^	13/21/28/18/20/16/0
tinnitus sound characteristics ^3^	71/13/20/9
intermittent vs. constant tinnitus ^4^	13/107
pulsation of tinnitus ^5^	10/09/99
loudness fluctuation ^6^	75/44
tinnitus frequency ^7^	38/55/22/2
maskability of tinnitus ^8^	82/25/12
somatic tinnitus ^9^	55/65
temporomandibular disorder ^10^	34/83
neck pain ^11^	73/47
other pain ^12^	52/68
tinnitus influenced by stress ^13^	97/23
subjective hearing problem ^14^	84/35
hearing aids ^15^	6/4/26/83
hyperacusis ^16^	76/35
headache ^17^	39/81
dizziness/vertigo ^18^	56/36

^1^: female/male; ^2^: right ear/left ear/both ears but worse on left/both ears but worse on right/both ears equally/inside the head/elsewhere; ^3^: tone/noise/cricket/other sound; ^4^: intermittent/constant; ^5^: yes, with the heart rate/yes, different from heart rate/no; ^6^: fluctuates/constant; ^7^: very high freq./high freq./medium freq./low freq.; ^8^: yes/no/do not know; ^9^: yes/no/do not know; ^10^: suffers from temporomandibular disorders/does not suffer from temporomandibular disorder; ^11^: suffers from neck pain/does not suffer from neck pain; ^12^: suffers from pain syndrome/does not suffer from pain syndrome; ^13^: stress influences tinnitus/stress does not influence tinnitus; ^14^: has hearing problem/does not have hearing problems; ^15^: right ear/left ear/both ears/none; ^16^: suffers from hyperacusis/does not suffer from hyperacusis; ^17^: suffers from headaches/does not suffer from headaches; ^18^: suffers from dizziness/does not suffer from dizziness.

**Table 2:** Comparison between the pandemic situation and the baseline. Mean values are given ± standard deviation. W: paired Wilcoxon test, *t*: paired *t*-test.

	*n*	Baseline	Pandemic	*p*-Value	Adjusted *p*	Cohen’s D	Hedge’s G
THI	112	41.52 ± 23.93	45.89 ± 23.47	0.013 (W)	0.222	0.130	0.129
Mini-TQ	104	10.18 ± 6.46	11.15 ± 6.38	0.027 (W)	0.297	0.100	0.099
MDI	102	13.06 ± 9.78	13.85 ± 9.78	0.543 (W)	1.000	0.019	0.019
BFI2 Extraversion	96	38.18 ± 7.73	37.56 ± 8.17	0.652 (t)	1.000	−0.021	−0.021
BFI2 Agreeableness	96	44.20 ± 6.10	44.98 ± 5.47	0.095 (W)	0.633	0.132	0.131
BFI2 Conscientiousness	96	45.91 ± 7.70	45.82 ± 7.85	0.643 (W)	1.000	0.064	0.064
BFI2 Neuroticism	96	35.97 ± 7.58	34.28 ± 8.70	<0.001 (t)	<0.01	−0.284	−0.281
BFI2 Openness	96	39.69 ± 7.67	40.49 ± 7.88	0.246 (t)	1.000	0.077	0.076

**Table 3:** Spearman correlations between tinnitus changes and pandemic-related burden. Tinnitus changes have been calculated by subtraction of the second time point (pandemic) from the first time point (baseline), meaning that positive change values indicate an increase of tinnitus-related distress. Higher values for the SOISES questionnaire items indicate disagreement with the statement. Therefore, the negative estimate for the correlation between grief and THI change indicates that more grief relates to higher THI scores. Please note that the variable stress is inversely coded. Results are visualized in Figure 2.

Pandemic-Related Stress Question (SOISES)	Tinnitus Questionnaire	Estimate (95% Confidence Interval)	*p*-Value	Adjusted *p*-Value
I have a feeling of grief	THI	−0.315 (−0.455, −0.161)	0.001	0.037
I have a feeling of grief	Mini-TQ	−0.333 (−0.177, −0.177)	0.001	0.029
I feel frustrated	THI	−0.196 (−0.360, −0.027)	0.038	0.852
I feel frustrated	Mini-TQ	−0.348 (−0.495, −0.185)	<0.001	0.023
I experience less stress than usual	THI	0.405 (0.243, 0.544)	<0.001	0.001
I experience less stress than usual	Mini-TQ	0.459 (0.301, 0.597)	<0.001	<0.001
I’m nervous	THI	−0.373 (−0.520, −0.208)	0.000	0.004
I’m nervous	Mini-TQ	−0.307 (−0.464, −0.132)	0.001	0.058

**Table 4:** Correlation analysis between tinnitus changes and personality traits during the pandemic. Tinnitus changes have been calculated by subtracting the value of the second time point (pandemic) from the first time point (baseline), meaning that positive change values indicate an increase of tinnitus-related distress.

Personality Trait	Tinnitus Questionnaire	Estimate (95% Confidence Interval)	*p*-Value	Adjusted *p*-Value
BFI2 Extraversion	THI	−0.353 (−0.518, −0.166)	<0.001	0.002
BFI2 Extraversion	Mini-TQ	−0.307 (−0.474, −0.121)	0.002	0.007
BFI2 Agreeableness	THI	−0.151 (−0.327, 0.030)	0.133	0.304
BFI2 Agreeableness	Mini-TQ	−0.160 (−0.335, 0.021)	0.112	0.256
BFI2 Conscientiousness	THI	−0.305 (−0.490, −0.103)	0.002	0.008
BFI2 Conscientiousness	Mini-TQ	−0.258 (−0.453, −0.045)	0.009	0.027
BFI2 Neuroticism	THI	0.617 (0.459, 0.741)	<0.001	<0.001
BFI2 Neuroticism	Mini-TQ	0.571 (0.401, 0.710)	<0.001	<0.001
BFI2 Openness	THI	−0.265 (−0.447, −0.070)	0.008	0.022
BFI2 Openness	Mini-TQ	−0.308 (−0.481, −0.118)	0.002	0.007

## Supplementary Materials

The following are available online at https://www.mdpi.com/2077-0383/9/9/2756/s1, Table S1. Spearman correlation of the THI change with the Items of the social isolation questionnaire; Table S2. Spearman correlation of the mini-TQ change with the Items of the social isolation questionnaire; Figure S1. Scatterplot of the THI change with the Big Five Personality Factors; Figure S2. Scatterplot of the mini-TQ change with the Big Five Personality Factors.
