# Supplementary material for: The Effect of Environmental Stressors on Tinnitus: A Prospective Longitudinal Study on the Impact of the COVID-19 Pandemic
Source: J Clin Med. 2020 Aug 26;9(9):2756. doi: 10.3390/jcm9092756 (PMC7565885; doi:10.3390/jcm9092756)

Supplemental Material

Table 1. Spearman correlation of the THI change with the Items of the social isolation questionnaire.

Item Nr	Item	Estimate	CI inf	CI sup	P-value	Adjusted p
q1	I have the feeling that I can maintain my social contacts well via internet, telephone and other means of communication.	0.066582	-0.12452	0.256154	0.485486	1
q2	I have the feeling that I can occupy myself meaningfully.	0.058055	-0.1301	0.243126	0.543176	1
q3	I fear for my own life.	-0.04109	-0.22645	0.147849	0.667048	1
q4	I fear for the lives of relatives and/or friends.	-0.00464	-0.19394	0.179859	0.961285	1
q5	I'm bored.	0.052165	-0.14893	0.252074	0.584904	1
q6	I'm concerned about my personal financial situation.	0.049869	-0.12668	0.224698	0.601552	1
q7	I have a feeling of sadness.	-0.31544	-0.45592	-0.15984	0.000705	0.036514
q8	I feel frustrated.	-0.196	-0.35974	-0.02684	0.038351	0.851705
q9	I fear infecting other people.	-0.00082	-0.19285	0.191987	0.993168	1
q10	I receive a lot of support from other people (e.g. family, friends, neighbours, social institutions ...).	0.058127	-0.13279	0.24503	0.542678	1
q11	I feel alone.	-0.17537	-0.34243	-0.00225	0.064387	1
q12	I sleep worse.	-0.18244	-0.35659	-0.00531	0.054189	1
q13	I have bad dreams.	-0.14582	-0.33076	0.046177	0.12501	1
q14	I feel less stressed than usual.	0.404665	0.243535	0.54645	9.63E-06	0.001497
q15	I feel helpless.	-0.2112	-0.38241	-0.03246	0.025396	0.658001
q16	I am afraid that my supplies (food, water, clothes, toiletries, medicine and whatever else I need to live) are not enough.	0.002769	-0.1925	0.198037	0.97688	1
q17	I feel guilty.	-0.1498	-0.33812	0.043848	0.114919	1
q18	I feel well informed about the current circumstances.	0.120794	-0.05457	0.291949	0.204558	1
q19	I'm nervous.	-0.373	-0.51935	-0.20752	5.11E-05	0.003976
q20	I feel lethargic.	-0.15663	-0.32903	0.022067	0.099112	1
q21	I have someone to talk to.	0.080128	-0.10342	0.261562	0.401001	1
q22	I have the feeling that even in quarantine I can keep good contact with my family and friends.	0.074802	-0.11778	0.256652	0.433132	1
q23	When I feel worse, I know what I can do or who to turn to.	0.102458	-0.07696	0.277637	0.282386	1
q24	I'm happy.	0.143291	-0.04176	0.31877	0.131754	1
q25	I'm certain that this situation will come to an end.	0.245603	0.063044	0.407866	0.00905	0.281377
q26	I'm confused.	-0.28185	-0.43661	-0.11501	0.002607	0.101323
q27	I currently consume more stimulants than usual (e.g. sweets, alcohol, nicotine, ...)	-0.12985	-0.31249	0.058278	0.172388	1
q28	I'm upset by the current situation.	-0.1091	-0.28948	0.075947	0.252159	1
q29	The uncertain future unsettles me.	0.028911	-0.1548	0.214122	0.762196	1
q30	I feel stigmatised.	0.051513	-0.12867	0.231633	0.58961	1
q31	I'm waiting for someone to call me or write to me.	0.011607	-0.1725	0.19568	0.903324	1
q32	I feel sufficiently well connected through telephone and internet.	-0.01684	-0.2115	0.175258	0.860113	1
q33	In my domestic isolation I have enough room for myself.	-0.01119	-0.20787	0.17767	0.906818	1

q34	Even in my domestic isolation I have enough social contacts.	0.059323	-0.13332	0.241143	0.534388	1
q35	I use social media (Whatsapp, Facebook, Twitter, Instagram, Reddit, ...) more than usual in the current situation.	-0.14989	-0.33391	0.041557	0.114702	1
q36	If I get infected, I am particularly at risk because I belong to a risk group (e.g. because of lung disease, immune deficiency, diabetes, old age).	0.017645	-0.17857	0.213583	0.853499	1
q37	I'm able to perform my usual activities (e.g. work, hobbies) as usual.	0.085886	-0.10776	0.277799	0.367907	1

Table S2. Spearman correlation of the mini-TQ change with the Items of the social isolation questionnaire

Item Nr	Item	Estimate	CI inf	CI sup	P-value	Adjusted p
q1	I have the feeling that I can maintain my social contacts well via internet, telephone and other means of communication.	0.090322	-0.10324	0.279272	0.36185	1
q2	I have the feeling that I can occupy myself meaningfully.	0.132734	-0.06626	0.323454	0.1792	1
q3	I fear for my own life.	0.015302	-0.17296	0.207201	0.877469	1
q4	I fear for the lives of relatives and/or friends.	0.064516	-0.1301	0.253	0.515265	1
q5	I'm bored.	0.040309	-0.15838	0.242567	0.684548	1
q6	I'm concerned about my personal financial situation.	0.055832	-0.12114	0.229783	0.573477	1
q7	I have a feeling of sadness.	-0.33289	-0.47754	-0.17646	0.000555	0.028752
q8	I feel frustrated.	-0.34785	-0.49504	-0.18768	0.000297	0.023064
q9	I fear infecting other people.	0.022523	-0.16359	0.212338	0.82047	1
q10	I receive a lot of support from other people (e.g. family, friends, neighbours, social institutions ...).	-0.0407	-0.2206	0.146909	0.681647	1
q11	I feel alone.	-0.14636	-0.3166	0.031268	0.138202	1
q12	I sleep worse.	-0.16715	-0.34721	0.018477	0.089896	1
q13	I have bad dreams.	-0.14563	-0.3359	0.054319	0.140183	1
q14	I feel less stressed than usual.	0.459443	0.295885	0.598574	9.3E-07	0.000145
q15	I feel helpless.	-0.2061	-0.37587	-0.01652	0.035813	0.795355
q16	I am afraid that my supplies (food, water, clothes, toiletries, medicine and whatever else I need to live) are not enough.	0.074155	-0.13027	0.274567	0.454385	1
q17	I feel guilty.	-0.19254	-0.38065	0.004497	0.050211	0.975714
q18	I feel well informed about the current circumstances.	0.04734	-0.14044	0.234404	0.633214	1
q19	I'm nervous.	-0.30747	-0.46366	-0.13228	0.001499	0.058247
q20	I feel lethargic.	-0.14463	-0.32111	0.041247	0.142957	1
q21	I have someone to talk to.	0.101478	-0.1016	0.298177	0.305357	1
q22	I have the feeling that even in quarantine I can keep good contact with my family and friends.	0.081479	-0.11843	0.279879	0.410935	1
q23	When I feel worse, I know what I can do or who to turn to.	0.112346	-0.06352	0.277933	0.256182	1
q24	I'm happy.	0.138863	-0.04491	0.317354	0.159774	1
q25	I'm certain that this situation will come to an end.	0.234645	0.035184	0.416601	0.016504	0.427622
q26	I'm confused.	-0.28804	-0.44171	-0.11741	0.003026	0.094084
q27	I currently consume more stimulants than usual (e.g. sweets, alcohol, nicotine, ...)	-0.06995	-0.26126	0.126702	0.480456	1
q28	I'm upset by the current situation.	-0.07983	-0.26302	0.108428	0.420505	1
q29	The uncertain future unsettles me.	-0.03473	-0.21841	0.153316	0.726367	1

q30	I feel stigmatised.	-0.02559	-0.21581	0.166258	0.796503	1
q31	I'm waiting for someone to call me or write to me.	0.089087	-0.09648	0.268159	0.368482	1
q32	I feel sufficiently well connected through telephone and internet.	-0.01906	-0.21971	0.18139	0.847671	1
q33	In my domestic isolation I have enough room for myself.	-0.03565	-0.23252	0.165095	0.719371	1
q34	Even in my domestic isolation I have enough social contacts.	0.017655	-0.18219	0.218642	0.858817	1
q35	I use social media (Whatsapp, Facebook, Twitter, Instagram, Reddit, ...) more than usual in the current situation.	0.003181	-0.19286	0.201397	0.974434	1
q36	If I get infected, I am particularly at risk because I belong to a risk group (e.g. because of lung disease, immune deficiency, diabetes, old age).	0.101624	-0.08604	0.28702	0.304655	1
q37	I'm able to perform my usual activities (e.g. work, hobbies) as usual.	0.040037	-0.16482	0.238025	0.686561	1

Figure S1. Scatterplot of the THI change with the Big Five Personality Factors

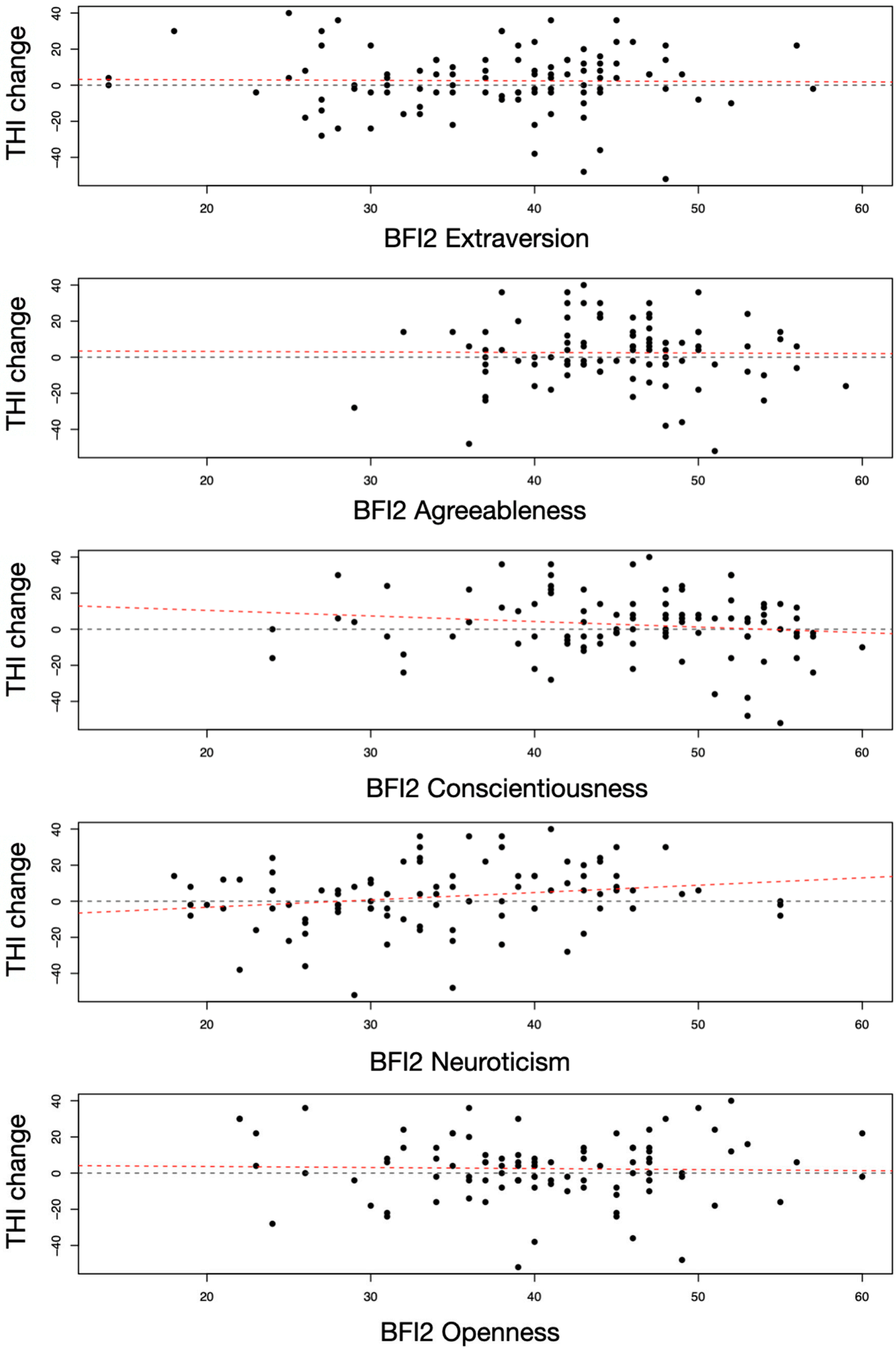

Figure S2. Scatterplot of the mini-TQ change with the Big Five Personality Factors

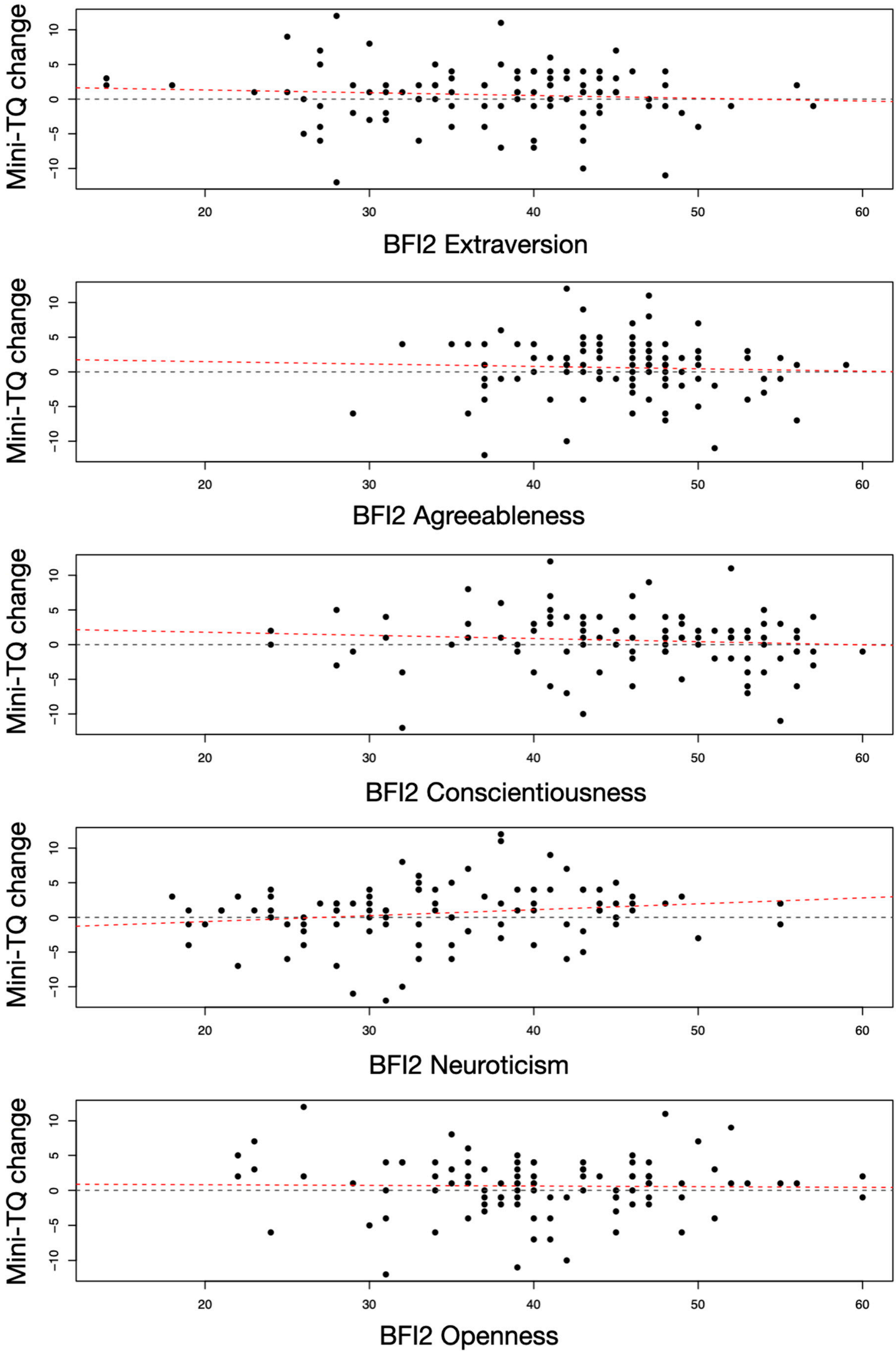

Supplement: Supplementary file 1 [file jcm-09-02756-s001.pdf]
